# Poly(vinyl alcohol) Nanocomposites Reinforced with CuO Nanoparticles Extracted by *Ocimum sanctum*: Evaluation of Wound-Healing Applications

**DOI:** 10.3390/polym17030400

**Published:** 2025-02-02

**Authors:** Shrishail Pattadakal, Vanita Ghatti, Sharanappa Chapi, Vidya G., Yogesh Kumar Kumarswamy, M. S. Raghu, Vidyavathi G. T., Nagaraj Nandihalli, Deepak R. Kasai

**Affiliations:** 1 Department of Chemistry, Faculty of Engineering and Technology, Jain University, Jakkasandra, Bengaluru 562112, Karnataka, India; shrish33jack@gmail.com (S.P.); vanitaghatti@gmail.com (V.G.); k.yogeshkumar@jainuniversity.ac.in (Y.K.K.); 2 Department of Physics, B.M.S. College of Engineering, Bengaluru 560019, Karnataka, India; 3 Department of Chemistry, Dayananda Sagar College of Engineering, SM Hills, Kumaraswamy Layout, Bengaluru 560011, Karnataka, India; vidyagopi5@gmail.com; 4 Department of Chemistry, New Horizon College of Engineering, Bengaluru 560103, Karnataka, India; raghuhassan2009@gmail.com; 5 Department of Chemistry, RNS Institute of Technology, Rajarajeshwari Nagar, Bengaluru 560098, Karnataka, India; vidyavathigt@gmail.com; 6 Critical Materials Innovation Hub, Ames National Laboratory, U.S. Department of Energy, Iowa State University, Ames, IA 50011, USA; nagaraj.nandi001@umb.edu

**Keywords:** green synthesis, biosynthesis, PVA, hemolysis, cytotoxicity, wound healing

## Abstract

This study focused on the synthesis of plant-mediated copper-oxide nanoparticles (OsCuONPs) via the sol–gel technique and the fabrication of OsCuONP-infused PVA composite films (POsCuONPs) utilizing the solvent casting method for wound-healing applications. The prepared OsCuONPs and nanocomposite films were characterized using UV–visible spectra, FTIR, SEM, XRD, TGA, water contact-angle (WCA) measurements, and a Universal testing machine (UTM) for mechanical property measurements. The UV and FTIR tests showed that OsCuONPs were formed and were present in the PVA composite film. Moreover, the mechanical study confirmed that there is an increase in the tensile strength (*T*_S_) and Young’s modulus (*Y*_m_) with 21.75 MPa to 32.50 MPa for *T*_S_ and 24.80 MPa to 1128.36 MPa for *Y*_m_, and a decrease in the % elongation at break (*E*_b_) (394.32 to 75.6). The TGA and WCA study results demonstrated that PVA films containing OsCuONPs are more stable when subjected to high temperatures and demonstrate a decreased hydrophilicity (60.89° to 89.62°). The cytotoxicity and hemolysis tests showed that the CuONPs-3 containing composite films (PVA/OsCuONPs with a wt. ratio of 1.94/0.06) are safe to use, have a good level of cell viability, and do not break down blood. This is true even at high concentrations. The study also discovered that cells moved considerably in 12 and 24 h (13.12 to 19.26 for OsCuONPs and 312.53 to 20.60 for POsCuONPs), suggesting that 60% of the gaps were filled. Therefore, the fabricated POsCuONP nanocomposites may serve as a promising option for applications in wound healing.

## 1. Introduction

Nanotechnology is a rapidly emerging field in which scientists and engineers develop new materials, modify existing ones, and synthesize new ones at the nanoscale. Certainly, the scientific community has begun to pay close attention to this area in the last several years [1,2]. With sizes ranging from 1 to 100 nm, nanoparticles (NPs) are essential in many fields, such as healthcare, agriculture, food science, energy, environmental clean-up, and gas adsorption [3,4,5]. The distinctive physicochemical properties, diminutive size, and consistent behavior of metal nanoparticles (MNPs) have attracted significant interest [6]. With such distinct characteristics, MNPs offer promising new avenues for use in cancer treatment, targeted drug delivery, wound healing, optical devices, catalysis, water purification, and antimicrobials [7,8]. Copper nanoparticles (CuNPs) are distinguished among various metal nanoparticles due to their applicability across many different industries. ZnO, CuO, Fe_3_O_4_, NiO, and bimetallic nanoparticles, as well as composites of these materials with polymers, have also been the subject of contemporary study [9,10,11]. However, substantial obstacles exist regarding their scale production and practical implementation, as the synthesis of these compounds is frequently labor-intensive, time-consuming, and expensive [12,13]. Diverse strategies can be employed to synthesize CuONPs, encompassing physical, chemical, and biological methods. In terms of scalability, cost-effectiveness, particle dispersion, and the controllability of particle size, each method presents distinct advantages and disadvantages. CuONPs are an ideal material for a wide range of applications due to their versatility, low cost, and ease of manufacturing.

An eco-friendly and efficient way to synthesize nanoparticles is the biosynthetic approach, which makes use of reducing agents such as biological microorganisms (e.g., fungus, algae, yeast, and bacteria), polysaccharides, and plant extracts (e.g., leaves, roots, and seeds). An increasing amount of interest has been directed toward medicinal plants due to their abundance of important phytochemicals that have a long history of usage in pharmaceuticals [14]. In particular, CuONPs produced from plants show promise as antibacterial and anticancer agents, which might lead to their use in many different biological settings. Polyphenols and proteins found in natural materials can be used as powerful reducing agents in green synthesis, allowing metal ions to be transformed into lower valence states. Green synthesis has several advantages over standard chemical and physical processes, including nontoxicity, pollution-free operation, cost-effectiveness, and ecological sustainability. In addition to exhibiting antioxidant and antibacterial characteristics, these compounds may be able to reduce metal salts to produce metal nanoparticles [15]. Furthermore, these phytochemicals enhance the reliability, biological compatibility, and eco-friendliness of the nanoparticles thus produced. Consequently, in comparison with traditional methods, the synthesis of nanomaterials from plants is advantageous due to its simplicity, reduced time requirements, and lower costs [16].

In comparison to other nanoparticles, CuONPs are optimal owing to their antibacterial, antifungal, antiviral, and anticancer properties. The remarkable properties of CuONPs as potent antibacterial, catalytic, anti-carcinogenic, and coating agents have been noted across various domains of biomedicine [17]. The strong bactericidal efficacy of CuONPs against diverse Gram-positive and Gram-negative bacteria renders them a compelling choice for wound dressings. Bioengineered CuONPs can revolutionize several fields, including healthcare, environmental protection, materials science, and energy, by offering more sustainable, efficient, and biocompatible solutions [18]. As antioxidants, antimicrobials, and anticancer agents, Cu and CuONPs have substantial applications [19,20]. Copper-oxide (CuO) nanoparticles may be readily produced by oxidizing Cu NPs. Tulsi (Ocimum sanctum) is an upright, heavily branching shrub that grows 30–60 cm tall and has hairy stems and simple, opposite green leaves that are highly aromatic. It is one of the most important and revered medicinal plants, and it is an integral part of our daily lives. Being a member of the Lamiaceae family of plants, the Tulsi plant is native to parts of China, Hainan Island, Taiwan, India, and North and Eastern Africa, and is often called the “queen of the herbs” or the “elixir of life” [21]. Tulsi has several medicinal uses, including the treatment of a wide range of infections (microbial, fungal, bacterial, viral, cancer, arthritis, chronic fever, infertility, and eye problems), along with its hepatoprotective, antispasmodic, analgesic, antiemetic, and heart-protective properties. Additionally, this medicinal herb has been shown to reduce blood glucose levels, making it an effective therapy for diabetes [22,23,24]. *Ocimum sanctum* has been utilized to reduce graphene oxide due to the presence of eugenol, ascorbic acid, and polyphenols, all of which have natural reducing capabilities. Similarly, cellulose/silver nanoparticle composite films have been prepared using *Ocimum sanctum* leaf extract as a reducing agent.

Polyvinyl alcohol (PVA) is a synthetic polymer that finds application in the medical field. In addition to being highly soluble in water, biodegradable, biocompatible, and showing little carbohydrate adsorption, PVA demonstrates chemical resilience [25]. The attributes of PVA render it advantageous in various prevalent domains, including healthcare, food packaging, manufacturing, the paper industry, and raw materials. The film-forming capabilities of PVA make it a versatile material that finds usage in wound dressings, soft contact lenses, eye drops, embolic filters, artificial cartilage, and meniscus, among other medical applications [26,27]. PVA is advantageous due to the reducing ability of secondary alcohol groups and it has been identified as a suitable host polymer for silver and other types of nanoparticles. The main objective of this study is to fabricate nanocomposites for wound healing by embedding different concentrations of CuONPs obtained by *Ocimum sanctum* leaf-extract mediation in PVA films. The current study encompassed the examination of UV, XRD, SEM, TEM, FTIR, TGA, mechanical, and water contact-angle properties of those prepared OsCuONPs and OsCuONPs/PVA nanocomposite films. Further, the proposed investigation also includes the assessments of cytotoxicity, hemolysis, and the wound-healing scratch assay. This research considers the physicochemical properties and efficacy of these materials in biological contexts, providing an in-depth analysis of their potential applications, especially as wound dressings.

## 2. Experimental

### 2.1. Materials and Methods

Poly(vinyl alcohol) (PVA) was procured from Central Drug House (CDH), New Delhi, India. Copper salt (CuSO_4_.5H_2_O mol. wt. 249.68) was procured from Fisher Scientific (Thermo Fisher Scientific India Pvt. Ltd. Delphi “B” Wing, Powai, Mumbai), and double-distilled water and NaOH were procured from Sigma Aldrich, Bangalore, Karnataka. The double-distilled water was used throughout the experiment.

#### 2.1.1. Preparation of Tulsi Extract

The *Ocimum sanctum* (Os) plant was collected from local Jakkasandra, 562112. The collected samples were washed with deionized water, dried at 30 °C in the oven, and powdered by a mortar. A total of 50 g of finely ground spice extract powder was dissolved in 100 mL of distilled water. The mixture was stirred for 12 h using a magnetic stirrer and kept in an oven overnight. The solution was filtered using Whatman No. 1 filter paper, and the filtrate was collected for further use.

#### 2.1.2. Preparation of Ocimum Sanctum Plant-Extract-Mediated CuO Nanoparticles (OsCuONPs)

OsCuONPs were prepared by dissolving 2.5 g of CuSO_4_.5H_2_O in distilled water (20 mL), and then a previously prepared 20 mL *Ocimum sanctum* plant extract was added to the CuSO_4_.5H_2_O solution. Both solution mixtures were left to be stirred with a magnetic stirrer for nearly 1 h. Later, this mixture was titrated against 1 N NaOH (1 g of NaOH dissolved in 250 mL distilled water) to obtain a precipitate. Again, the solution was stirred with a magnetic stirrer for half an hour and the precipitate was centrifuged for 10 min at 1050 rpm. The upper clear solution was discarded and the precipitate was washed with distilled water followed by 2% alcohol wash. The supernatant solution was neglected, the precipitate was collected in the crucible, and the extract were calcined (350 °C) in the oven for 4 h. The black crystals were powdered and stored for further preparations.

#### 2.1.3. Green Synthesis of CuO Nanoparticle (OsCuONP)-Doped PVA Composite Films

**Scheme 1:**
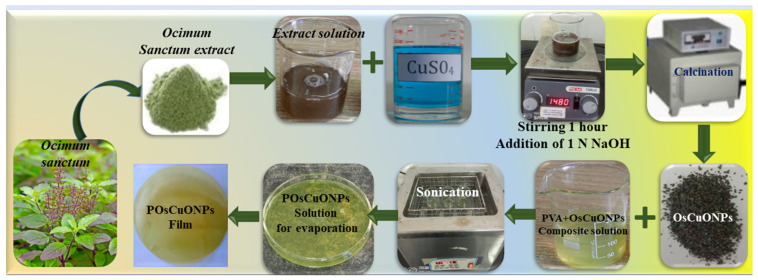
A schematic representation of the synthesis of PVA and OsCuONP-doped POsCuONP composite films.

To prepare pure PVA films, 2 g of PVA was dissolved in distilled water with constant stirring for 2–3 h, and a clear PVA solution was poured into the Petri dish and allowed to dry at room temperature. Similarly, different weight ratios of OsCuONPs were taken (0.02 g, 0.04 g, and 0.06 g) and dissolved in 20 mL of distilled water and kept for sonication for 1 h to ensure the complete dissolution or dispersion of nanoparticles. Later, different ratios of OsCuONP solutions were mixed with 80 mL of PVA stock solution and, again, sonicated for 1 h. The PVA/OsCuONP (POsCuONP) nanocomposite solutions were poured onto the Petri dishes and allowed to dry at room temperature. We finally peeled off the prepared POsCuONP nanocomposite films and stored them in plastic pouches for further property analysis. Table 1 shows the wt. ratios and the PVA/OsCuONPs of different samples. Schematic representation of PVA and OsCuONPs doped POsCuONPs composite films syn-thesis as shown in Scheme 1.

### 2.2. Characterizations

#### 2.2.1. Thickness and Appearance

Film thickness was measured using a Mitutoyo dial thickness gauge. Thickness measurements were taken over multiple places, and an average was taken. The thickness of all blend films was found to be around 0.19 mm.

#### 2.2.2. UV–Visible Spectroscopy

The formation of OsCuONPs was confirmed by using a UV–Vis spectrophotometer, specifically a two-beam T80 UV–Vis spectrophotometer, which recorded spectra between 200 nm and 800 nm.

#### 2.2.3. Fourier Transfer Infrared Spectroscopy (FTIR)

To verify the formation of OsCuONP and POsCuONP composite films, attenuated total reflection Fourier transform infrared (FTIR) spectroscopy was employed (Prestige 21, Shimadzu, Japan). Measurements were taken within the range of 400–4000 cm^−1^ with a resolution of 4 cm^−1^.

#### 2.2.4. Mechanical Properties

A Universal testing machine (UTM, LLOYD Instruments, India) was employed for the purpose of determining the mechanical properties. The tensile strength (*T*_S_), elongation at break (*E*_b_), and Young’s modulus (*Y*_m_) were tested in compliance with ASTM D882-91. The extension grips of the testing equipment were utilized to secure the 2.5 cm × 10 cm produced rectangular film samples. The film samples were run through the machine at a speed of 5 mm/min while the temperature was kept at room temperature.

#### 2.2.5. Scanning Electron Microscopy

The morphology and formation of OsCuONP and POsCuONP composite films were examined using an SEM (JEOL JSM-6360) (Jayanagar 7th block (west), KR Road, Bangalore, India) at an accelerating voltage of 10 kV. To prevent charging by powerful electron beams, all test materials were sputter-coated with a conductive coating of gold prior to the experiment. The film specimens were affixed to the sample holder using double-sided adhesive carbon tapes.

#### 2.2.6. Transmission Electron Microscopy

The presence of nanoparticles and microstructures of the POsCuONP nanocomposite films were analyzed by using HRTEM (Thermofisher, Talos F200 S, 200 KV, FEG, CMOS Camera 4K × 4K).

#### 2.2.7. X-Ray Diffraction

Ragaku D/Max-IIA X-ray diffraction (Tokyo, Japan) was used to examine the crystal structure of the OsCuONPs, crystallinity, and the impact of crystallinity on PVA composite films. A Cu-K*_α_* source operating at 30 kV (a wavelength of 1.5405 Å) was employed to produce the radiation. Applying a current of 20 mA, the samples were scanned at a rate of 2°/min from 0° to 80°. The determination of crystallinity was made using the data on diffracted intensity. For the purpose of evaluating the crystallite size (P), Scherrer’s Equation (1) was applied to the observed WAXD data.

(1) $$P=\frac{k\lambda }{\beta cos\theta }$$

where k = 0.9 (a constant is given for the shape of the crystal and the Miller index of the reflecting crystallographic plates as well as the crystallite shape); θ indicates the Bragg’s angle, λ = 1.5406 Å, the wavelength of X-ray radiation, and β is the width taken at half the maximum intensity of the reflection in radians.

#### 2.2.8. Thermogravimetric Analysis

In order to study how the prepared composite films degraded, thermogravimetric analysis was performed using SDT Q600 V20.9TA devices. Samples weighing ~6–8 mg were heated at a rate of 10 °C/min in an inert nitrogen atmosphere until they attained 800 °C.

#### 2.2.9. Water Contact Angle

The hydrophobicity and hydrophilicity of the composite films were evaluated by measuring the contact angle of the produced films. The contact angle of the water was measured using the SEO Phoenix instrument. Every test was carried out at room temperature with a 7 µL size drop. Contact-angle measurements were performed at nearly five different locations using the corresponding software.

#### 2.2.10. Cytotoxicity

The cytotoxicity tests were performed on the prepared composite films. The first step in analyzing cytotoxicity was to trypsinize the cells and then aspirate them into a 15 mL centrifuge tube. A cell pellet was produced by centrifugation at a speed of 300× *g*. Approximately 10,000 cells were suspended in 200 µL of Dulbecco’s Modified Eagle Medium (DMEM) to regulate the cell count. Approximately 200 µL of the cell suspension was added to each 96-well microtiter plate. The plates were then incubated at 37 °C in a 5% CO_2_ environment for approximately 24 h. Subsequently, the spent media was removed. Then, 200 µL of various test drug concentrations (20, 40, 60, 80, and 100 μg/mL) were applied to the corresponding wells. After placing the plate in an incubator set at 37 °C with a 5% CO_2_ environment for 24 h, the drug-containing fluid was withdrawn and the plate was re-incubated. Finally, 100 μL of media containing 10% MTT reagent was added to every well until the total concentration reached 0.5 mg/mL. The plate was then incubated at 37 °C in a 5% CO_2_ environment for three hours. Afterward, 100 μL of DMSO solubilization solution was introduced to the withdrawn culture media, and the plate was gently agitated in a rotary shaker to dissolve the formazan that had formed. Two wavelengths were used by a microplate reader to measure the absorbance 570 nm and 630 nm. By removing the blank and background from the amount of growth, inhibition was carried out using the dose–response curve for the cell line. The IC50 was then determined by finding the concentration of the test medication that inhibited cell growth by 50%.

#### 2.2.11. Assay for Blood Compatibility (Hemolysis)

A healthy participant’s blood (5 mL) was used to obtain data for the hemolysis assay, which assessed the amount of hemolysis produced by nanocomposite films when exposed to fresh human blood. The blood samples underwent centrifugation. The leucocytes and plasma were gradually separated and then mixed at 3000 rpm for 10 min while the mixture remained at room temperature. Normal physiological salt water was washed three times with 0.9% NaCl to remove red-blood-cell pellets. The blood sample became transparent after centrifugation at 3000 rpm for 10 min and cleaning. After being soaked in normal saline, 1 cm × 1 cm films were left to incubate at 37 °C for 24 h. The following step was to add 200 μL of blood and 200 μL of incubated test samples, followed by 30 min of incubation at 37 °C. In order to prevent hemolysis, 4 mL of physiological saline was added to the samples and left for 60 min. Hemolysis of red blood cells in distilled water and sterile physiological saline, respectively, were used as positive and negative controls, without film samples. After that, the absorbance of the supernatant at 540 nm was measured by centrifugation at 3000 rpm for 10 min. A measure of hemolysis percentage was determined by using the below formula.

Hemolysis (%) = (test sample abs − negative control abs/positive control abs − negative control abs) × 100

#### 2.2.12. Wound-Healing Scratch Assay (Cell Migration)

The cells were trypsinized and aspirated into a 5 mL centrifuge tube. The cell pellet was obtained by centrifugation at 300× *g*. The cell count was adjusted using DMEM. To each well of the 12 well plate, 1 mL DMEM containing 100 μL of the cell suspension was added and the plate was incubated at 37 °C and 5% CO_2_ atmosphere for 24 h to reach ~100% confluence as a monolayer. Without changing the medium, the monolayer was gently and slowly scratched with a new 200 μL pipette tip across the center of the well. While scratching across the surface of the well, the long-axial of the tip should always be perpendicular to the bottom of the well. The resulting gap distance, therefore, equals the outer diameter of the end of the tip. The gap distance can be adjusted by using different types of tips. The scratch was made in a straight line in one direction. After scratching, we gently washed the well twice with medium to remove the detached cells. Then, we washed the cells twice with 1× PBS and replenished the well with fresh medium. The PBS was aspirated and test concentrations from the stock of test drugs were added to the respective wells with 1 mL of fresh medium. The photos of the scratched monolayer were taken at different time intervals, 0 h, 6 h, 12 h, and 24 h, with the subsequent incubation of the plate at 37 °C and a 5% CO_2_ atmosphere for 24 h time intervals. The gap distance can be quantitatively evaluated using MagVision Software(version 7.2) by measurement calibration at 4X resolution. To determine the migration rate, we used the following formula:

*R*_m_ = (*W*_i_ − *W*_t_)/*T*

*R*_m_—Rate of cell migration (µm/h)
*W*_i_—Initial wound width (µm)
*W*_t_—Final wound width (µm)
*T*—Duration of migration (hour)

Percentage of wound closure = Initial wound diameter-Final diameter ÷ Initial diameter × 100

## 3. Results and Discussion

### 3.1. UV–Visible Spectroscopy

**Figure 1:**
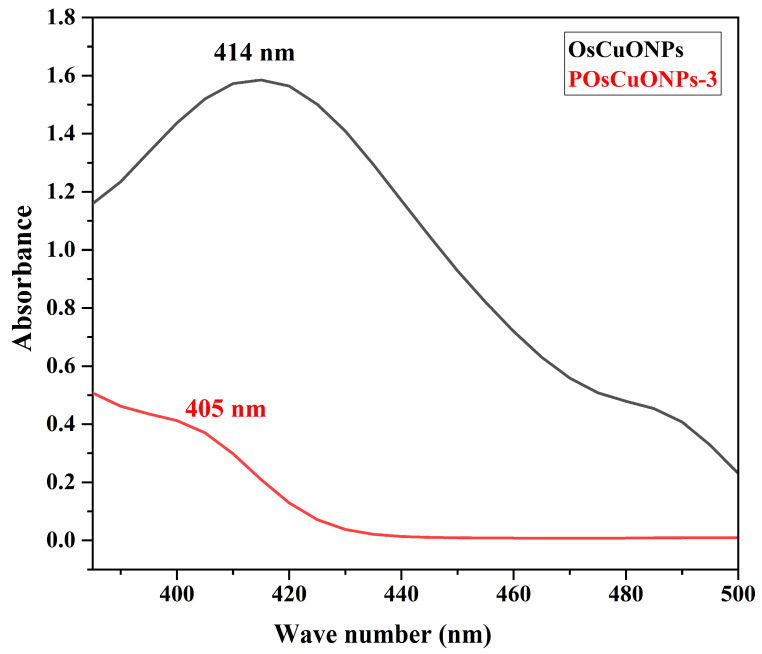
UV–visible spectra of OsCuONPs and POsCuONPs nanocomposite films.

The formation of OsCuONPs and their interaction in the POsCuONP nanocomposite are presented in Figure 1. The peak at 414 nm, seen in Figure 1, was attributed to the effective bio-reduction of CuSO_4_ to OsCuONPs. There is a robust relationship between the absorbance spectra of green-produced OsCuONPs and earlier findings [28]. V Sujatha et al. have reported that the ultraviolet–visible spectra of the water-based *S. foetida* leaf extract shows two distinct bands at 312 nm, which correspond to the π→π* transition, and at 370 nm, which correspond to the *n*→π* and π→π* transitions. The results of the UV–visible examination showed that the plant extract contained phenolic and flavonoid chemicals, which are characterized by these absorption bands [29]. However, there is a strong correlation between the absorbance spectra of the green-produced OsCuONPs and previous reports [30,31]. A transition from blue to dark brown in the solution color was seen during synthesis, signifying the creation of copper-oxide nanoparticles. It was found that the synthesis of OsCuONPs was indicated by a change in color, which occurred when aqueous leaf extract of *Ocimum sanctum* was used as a green reducing agent to reduce Cu ions into OsCuONPs. A similar finding has been confirmed in the study of Punniyakoti et al. [32]. Another possible explanation for the color shift is the interaction between incoming photons and metal nanoparticles that transport electrons [33]. Due to the addition of OsCuONPs to the PVA film, it was observed that the absorption peaks in the range of 405 nm slightly decreased. The establishment of a strong intermolecular interaction among the OsCuONPs lowers the wavelength with the incorporation of nanoparticles.

### 3.2. Fourier Transfer Infrared Spectroscopy (FTIR)

**Figure 2:**
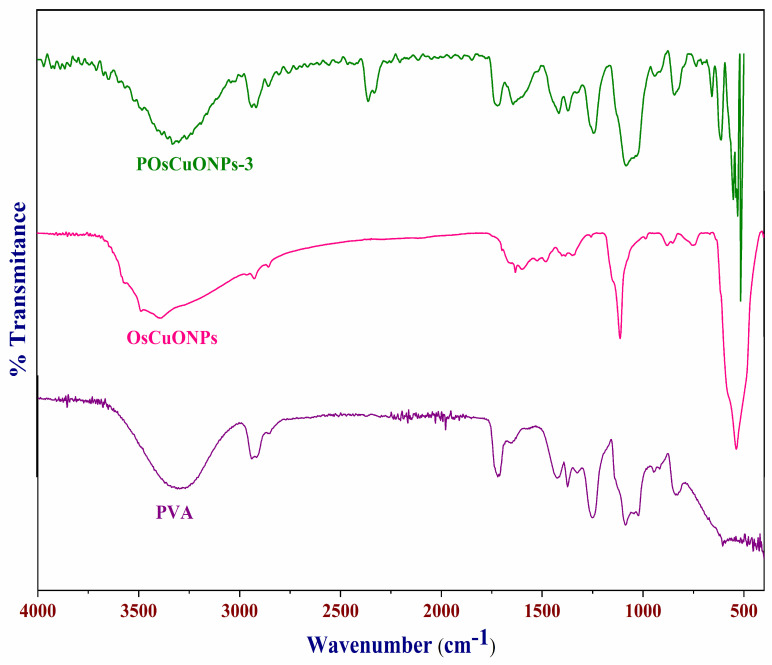
FTIR spectra of pure PVA, OsCuONPs, and POsCuNOP composite films.

To assess the potential interactions among the CuONPs and PVA matrix, we recorded the FTIR spectra of pure PVA and OsCuONP-filled PVA nanocomposites. Figure 2 summarizes the FTIR spectra of POsCuONP-3 and PVA. The stretching vibrations of the -OH groups in pure PVA were detected at 3320 cm^−1^, while the asymmetric and symmetric stretching of the C-H alkyl groups in the material were identified at 2936 and 2862 cm^−1^, respectively. In addition, the transmittance peak at 1732 cm^−1^ is attributed to the C=O stretching of the acetate group in PVA. The peaks at 1432 cm^−1^ and 1092–1154 cm^−1^, respectively, are peaks caused by the presence of the C–H bending of the CH_2_ bending and the acetyl C–O stretching of PVA. A peak at 834 cm^−1^ indicates that the C-C band of PVA is vibrating [34,35]. The CuONPs’ FTIR spectra display peaks around 3394 cm^−1^, which are ascribed to the hydrogen bound -OH groups linked to the phenols, alcohols, and -NH of amides, which are aromatic compounds [36]. The OsCuONPs’ FTIR spectra display peaks around 3394 cm^−1^, which are ascribed to the hydrogen bound -OH groups linked to the phenols, alcohols, and -NH of amides, which are aromatic compounds [37]. The peaks at 1630 cm^−1^ in the extract can be explained by the presence of amide linkages in proteins and enzymes, primary and secondary amines, and other similar compounds [38].

Confirmation of the synthesis of OsCuONPs was provided by the existence of a band at 535 cm^−1^, which is indicative of Cu-O vibrations. In order to prevent agglomeration and act as a capping agent, FTIR analysis confirms the presence of phenolic compounds and proteins involved in synthesis. The presence of unattached carboxylic and amino groups might be a reason why the CuONPs are stable. One probable process via which OsCuONPs can be formed in films is when bioactive substances stimulate a reduction in the metal precursor. After reacting with bioactive chemicals, copper hydroxide is formed when copper sulfate (CuSO_4_) combines with water (-OH). The bioactive chemicals included in the spice extract serve as reducing agents, passivating the contacts for stabilization, and acting as capping agents, which leads to the synthesis of OsCuONPs [39].

### 3.3. Mechanical Properties

**Figure 3:**
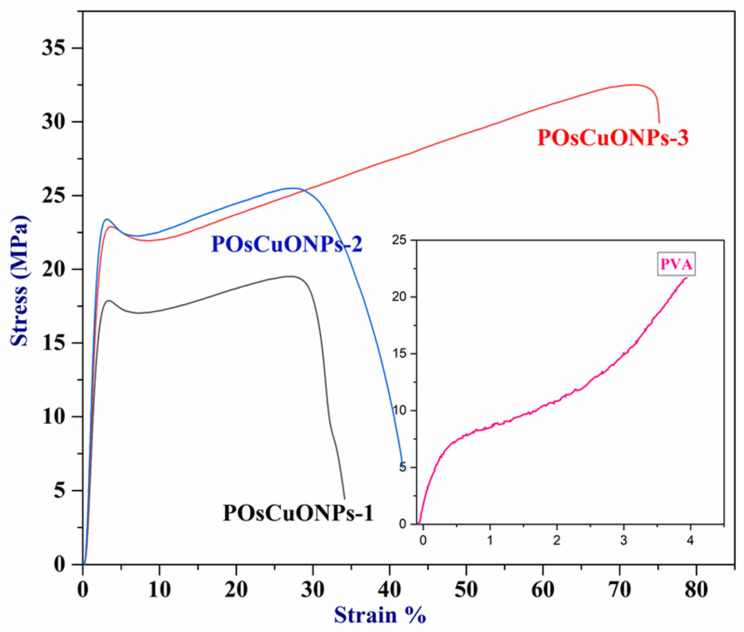
Stress–strain curve of pure PVA and POsCuNOP composite films.

Wound-dressing materials must possess sufficient mechanical strength to adequately cover and safeguard the wound, which is their primary role. To facilitate patient mobility and accommodate different joints and folding areas, wound dressings are designed to be as flexible as possible. Materials with a high modulus of elasticity and tensile strength are considered flexible. In order to assess the contribution of CuO nanoparticles to mechanical properties, pure PVA and POsCuONP composite films were analyzed with UTM. The results of the mechanical studies are summarized in Table 2, and the stress–strain curve is presented in Figure 3. The mechanical properties of the PVA nanocomposite films showed a *T*_s_ of 21.705 MPa, a *Y*_m_ of 24.483 MPa, and an %*E*_b_ of 394.327. In comparison with pure PVA, elongation at break has drastically decreased in POsCuONP composite films. This could possibly be due to the dominancy maintained by the crystallinity in the spice extract as well as OsCuONPs. However, by increasing the OsCuONP content in the PVA film, the values of elongation at break increased from 34.13 to 75.16%. According to these findings, molecular interaction inside the polymeric matrix would be enhanced with the addition of nanoparticles. The hydroxyl groups of PVA and OsCuONPs form stronger bonds by chelation at increased nanofiller content, which increases the bond strength and ultimately increases the final stress and elongation at break. A similar observation was recorded by the authors in the literature [40]. Better tensile strength and less elongation at break (*E*_b_) are the two most fundamental indicators that support the idea that nanoparticle reinforcing is effective [41]. It is clear from Table 2 that the tensile strength of the nanocomposites is significantly greater than that of the pure PVA film.

The strong interaction among the PVA chains and CuONPs is greatly responsible for the improved mechanical properties. Nanoparticles and PVA chains exhibit significant interfacial adhesion, which enhances the resulting nanocomposite’s tensile strength. This shows that the OsCuONP nano-filler is distributed more evenly, creating a big surface area that helps the composite and OsCuONPs stick together better [42]. Also, the mechanical properties of POsCuONP nanocomposites depend on how evenly the nanoparticles are spread out in the PVA polymer matrix and how much the OsCuONPs and PVA film interact with each other.

### 3.4. Scanning Electron Microscopy Studies

**Figure 4:**
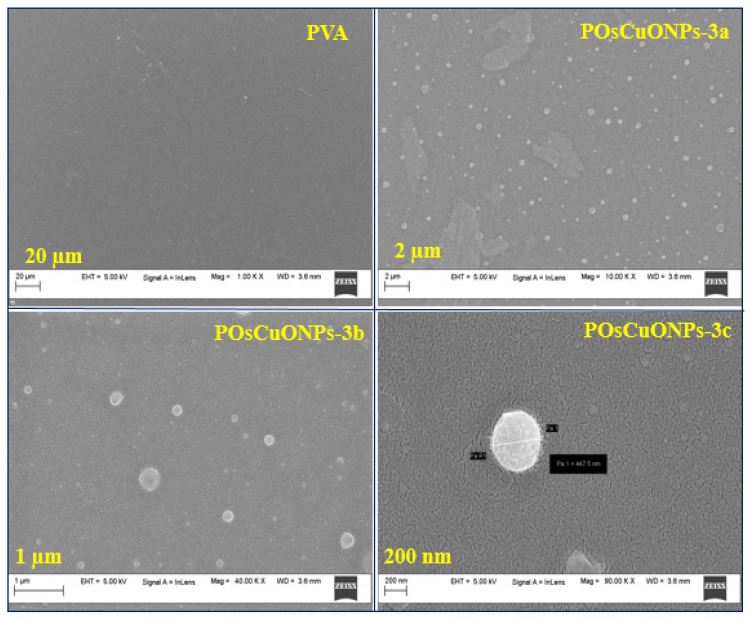
SEM micrographs of pure PVA and POsCuNOPs-3 composite films with different magnification values.

The main parameters that determine the shape of the films are the size and distribution of the dispersed particles, the solvent utilized, and the nature of the solvent evaporation process [43]. It is worth noting that interactions among PVA and OsCuONPs were defined by the dispersion of particles and distribution inside the polymer matrix. An SEM was used to investigate the morphology and existence of OsCuONPs in PVA composite films. Additionally, EDS was employed to examine the chemical composition and distribution of the additives in the films of nanocomposites. Figure 4 displays scanning electron micrographs of pure PVA and POsCuONP-3 at different magnifications. Pure PVA film has a uniformly smooth appearance [44,45]. Since the PVA matrix serves as a support matrix for the nanocomposites’ many components, the SEM micrographs show that the OsCuONPs (in POsCuONP-3) are uniformly distributed throughout the matrix [46,47]. Figure 4 shows that the PVA structure contains evenly dispersed nano-sized particles made from plant extracts that were converted into a polymer film. White, spherical particles of varying sizes were determined to be the structures produced by the significant interaction between the PVA and OsCuONPs, as well as by the solvent evaporation that occurred during drying [47]. In this study, the SEM images and free ImageJ program were used to determine the dispersion and particle size of the biosynthesized OsCuONPs, as carried out in the case of some previous studies [48,49,50,51,52].

### 3.5. Transmission Electron Microscopy

**Figure 5:**
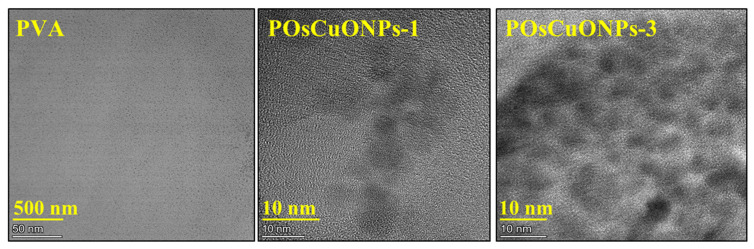
TEM micrographs of pure PVA, POsCuNOPs-1, and POsCuNOPs-3 composite film.

Figure 5 shows the presence of nanoparticles and microstructures of the nanocomposite films as further described by the transmission electron microscopy (TEM) images. The presence of OsCuO nanoparticles that have been green-synthesized has a notable impact on the composites’ structural and morphological characteristics, according to the findings. A uniform casting solution and excellent film formation are indicated by the smooth surface of the pure PVA film in Figure 5. On the other hand, the TEM image of the nanocomposite films shows that the nanoparticles are effectively embedded in the polymeric matrix regardless of the concentration of OsCuO. It is probably easier for OsCuO particles to create preliminary bonding with the matrix when hydroxyl groups are present in the polymer structure. The higher mechanical stability and strength of the resulting nanocomposite films are thought to be due, in part, to the OsCuO particles’ capacity for self-assembly and their interactions with the polymer.

### 3.6. X-Ray Diffraction

**Figure 6:**
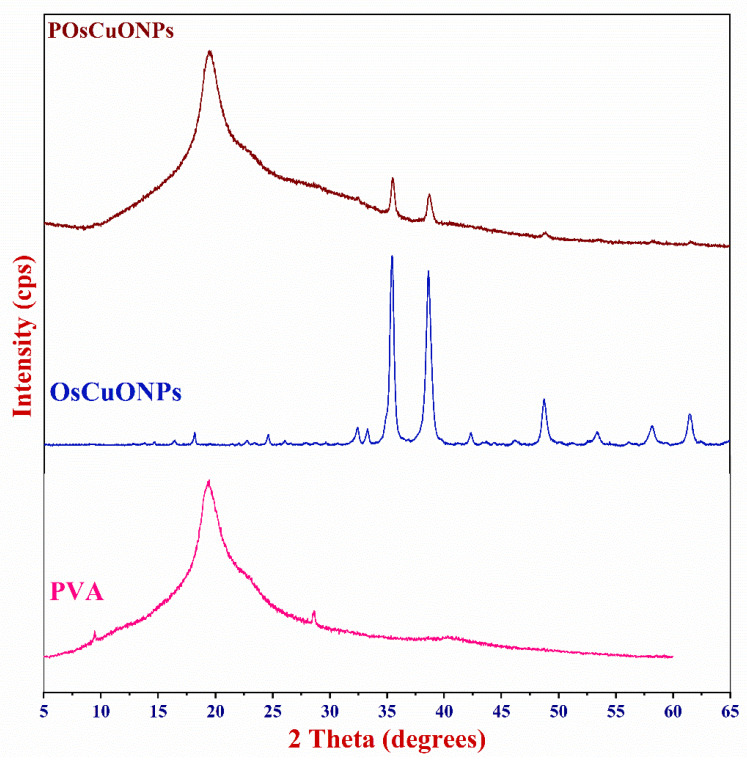
X-ray diffractogram of pure PVA, OsCuONP, and POsCuONP composite films.

The X-ray diffractogram was used to examine how OsCuONPs affected the crystallinity of the PVA films. Figure 6 shows the XRD patterns of the pure PVA, OsCuONP, and POsCuONP composite films obtained with a 2°/min scanning rate, spanning 10° ≤ 2*θ* ≥ 80°. Diffraction peaks at 2*θ* = 19.43° have been observed in pristine PVA, which may be due to the crystalline structure of the material caused by the strong intermolecular hydrogen bonds found in the PVA chains, which are in accordance with the findings from Darwish et al. 2022 [53]. The addition of plant-mediated OsCuONPs to the PVA matrix was shown to reduce the intensity of the PVA peak at 2*θ* = 19.50°. Figure 6 shows that the structure of the PVA chain was disrupted due to interactions between the PVA and other substances, which increased the amorphousness and caused the intensity to drop. The XRD findings show that the prepared OsCuONPs were crystalline and had a certain phase orientation. The X-ray patterns of OsCuONPs had peaks at 2*θ* values of 32.36°, 33.27°, 35.48°, 38.62°, 42.50°, 45.98°, 48.84°, 53.45°, 58.16°, 61.47°, 66.08°, 68.01°, 72.60°, and 75.11°, which were tagged as the (110), (–111), (111), (–202), (020), (202), (–113), (–311), (220), (311), and (004) planes, respectively. The findings are in agreement with the CuONP standard data card (JCPDS-45-0937), indicating that the NPs generated are crystalline with an average size of 34 nm. Comparable findings were reported for copper-oxide nanoparticles prepared using *Malva sylvestris* leaf extract [54,55,56]. The absence of any further diffraction peaks indicates the presence of a pure CuONP phase.

Also, the XRD pattern of the CuONP-filled PVA composite films shows that the 2*θ* values are higher than in the pure PVA film. For example, POsCuONP-3 showed 2*θ* values at 9.66°, 19.97°, 23.48°, 40.56°, and 62.23°. Similarly, in the POsCuONPs-3 composite films, additional peaks were seen with higher 2*θ* values. As a result, we could begin to speculate whether the POsCuONPs-3 produced within the films affected the PVA packing, which in turn increased the crystallinity. The nanocomposite films showed a distinct peak of the OsCuONPs in POsCuONPs, suggesting a significant dispersion of OsCuONPs in the PVA polymer matrix [57]. The effective integration of nanoparticles into the polymer matrix and the purity of the phases are confirmed by these observations.

### 3.7. Thermogravimetric Analysis

**Figure 7:**
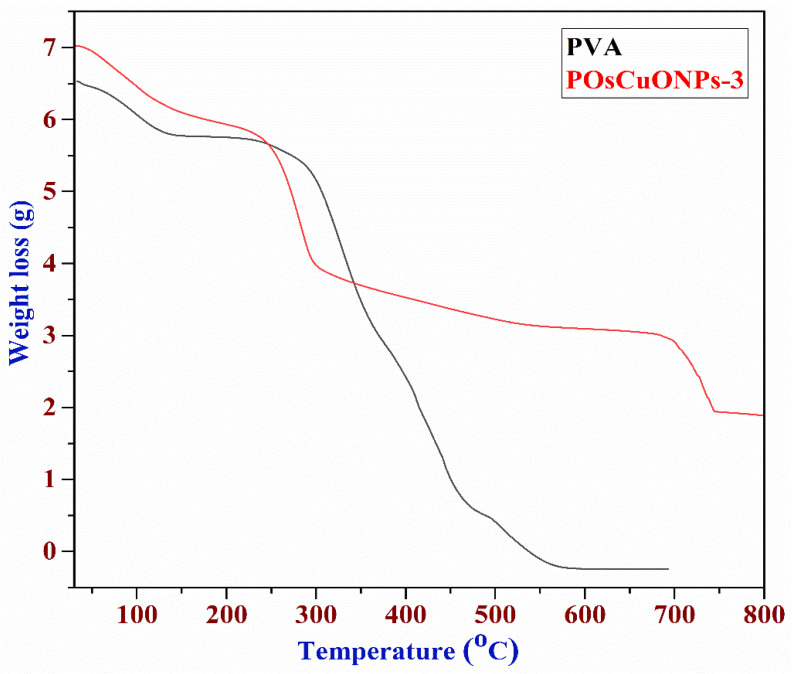
Thermogravimetric images of PVA and POsCuONPs-3 composite films. (red: composite; black: pure PVA film).

Figure 7 illustrates the results of the thermogravimetric analysis performed on the PVA, OsCuONPs, and POsCuONPs-3 composite films to assess their thermal stability. The degradation of PVA was shown by the TGA curve in three separate stages. The loss of absorbed moisture caused the first stages of deterioration to occur between 38 and 147 °C. The thermal degradation PVA chain was responsible for the most significant weight loss during the second degradation, which occurred between 147 and 277 °C. The release of by-products from the second degradation stage is associated with the degradation that occurs in the third step, which occurs between 277 and 586 °C. One study found that the lateral group acetate leaves PVA around 300 °C, which leads to maximum weight loss. The actual degradation of the PVA polymeric network happens between 350 °C and 450 °C. The results of the thermogravimetric study demonstrated that PVA films containing OsCuONPs that were green-synthesized are more stable and durable when subjected to high temperatures [58]. Perhaps the interaction between the nano CuO and the hydroxyl groups of the polymer matrix is responsible for the improved thermal stability seen when OsCuONPs are added to PVA. Another possibility is that the nanoparticles in suspension, like OsCuONPs, can stop the flow of degradation products, which would delay the start of degradation [59]. One study asserted that adding nanocomposites to polymers increased their heat stability, and many other organizations have investigated this topic [60]. It should be noted that many studies have yielded mixed results for the thermal stability of PVA/TiO_2_ and PVA/ZnO. Some investigations found improved thermal stability as a result of reduced molecular mobility and gas flow prevention during the degradation process. However, the observed behavior can be linked to the nature of the nanoparticles investigated, as the metallic atoms in them can act as degradation promoters in some of these composites.

### 3.8. Water Contact Angle (WCA)

**Figure 8:**
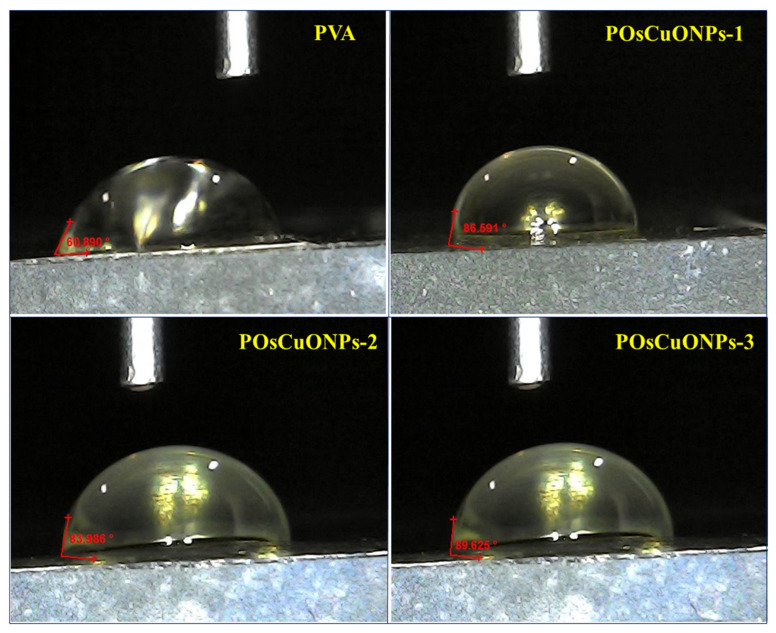
Contact-angle measurement of pure PVA and POsCuNOP composite films.

The wettability of the prepared PVA and POsCuONPs composite films was examined via water contact measurement (Sessile drop method). The contact angle of the neat pristine PVA and POsCuONPs composite films is shown in Figure 8. The contact angle of pure PVA was found to be 60.89°, indicating PVA is hydrophilic. There are noticeable shifts in the contact angle for POsCuONP composite films. In the case of green-synthesized OsCuONP-added PVA films, they exhibited increased contact angles of 86.59° (POsCuONP-1) and 92.33° (POsCuONP-3) for the composite films. As expected, adding OsCuONPs to the PVA film made it less hydrophilic and more hydrophobic. The present findings were in good agreement with the mechanical property and FTIR studies. The higher contact angle shows that the films were becoming more hydrophobic, but not completely. PVA demonstrates intrinsic hydrophilicity due to the abundance of -OH groups on its backbone; this is seen by its WCA of 60.89°. The presence of OsCuONPs in the PVA was shown to enhance the WCA, which may be explained by the fact that the nanocomposite surfaces interact with fewer hydroxyl moieties, leading to a decrease in hydrophilicity. By reducing the number of vacant spaces in the matrix and cross-linking its congeners, OsCuONPs may improve the hydrophobic characteristic of the composite films by making the polymer chains less mobile.

This phenomenon may be amplified even more, according to the SEM data, i.e., the increased surface roughness of the produced films. The increased surface roughness restricts the wetting liquid’s ability to interact with and penetrate the surface of the nanocomposite [61].

### 3.9. Cytotoxicity

**Figure 9:**
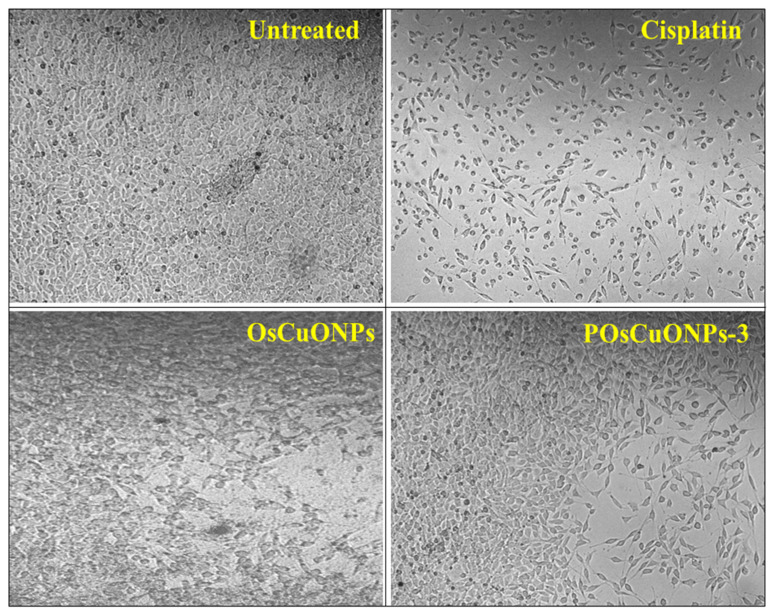
Cytotoxicity images of untreated, cisplatin, OsCuONP, and POsCuNOP composite films.

Considering the cytotoxicity of samples is essential, since the biocompatibility of the material is vital for its potential medical applications. Using the L929 fibroblast cell line, we conducted cell viability tests to prove that OsCuONPs and POsCuONPs-3 films are cytocompatible. The samples with the highest concentration, such as POsCuONPs-3 composite films and OsCuONPs, were tested to confirm the non-toxicity and cell viability. The films for cell growth using MTT assay were performed at 24 h to predict the propensity of films to be recommended for the applications of wound healing. The results of the cell viability and cytotoxicity of OsCuONPs and POsCuONPs-3 composite films are shown in Figure 9 and Table 3. Typically, PVA is thought of as being biocompatible and exhibits minimal cytotoxicity. Its biocompatibility and lack of toxicity make it ideal for usage in the biomedical field [62]. The results presented in Figure 9 and Table 3 show that untreated cells have 100% viability, indicating cells are alive and healthy. In addition to the usual drug cisplatin, the study found that the cells remained alive after treatment with the chemical at a certain dose.

Furthermore, after 24 h of treatment, OsCuONPs and POsCuONPs-3 samples with the L929 cell line and the test drugs’ IC50 values were found to be 58.34 µg/mL (OsCuONPs) and 66.65 µg/mL (POsCuONPs-3), respectively. This indicates that when cells are exposed to the samples being tested, they require a concentration of 58.34 µg/mL and 66.65 µg/mL to inhibit the viability of the cell by 50%. The decrease in the cell viability of the POsCuONPs-3 could be due to the diversity in the chemical influence that slightly affected the biological properties of the film. These results are consistent with those of Date et al. and Rukan Genc et al., who also observed similar results. In light of what they discovered, the cell vitality for PVA and 0.05YCD/PVA films at 500 mg/L was considerably decreased at 48 h (*p* < 0.05 (*)). However, at all concentrations tested, the cell viability for the other 0.05CD/PVA films (red, blue, and green) remained unaffected [63,64]. Statistical hypothesis testing uses the *p*-value as a metric. The *p*-value represents the likelihood of obtaining outcomes at least as extreme as those observed, assuming the null hypothesis is correct. It is vital to realize that the cytotoxicity could fluctuate according to the particular formulation, the study’s cell types, and the experimental methodology. Lower quantities of OsCuONPs and POsCuONPs-3 films were shown to be harmless to the tested cell line. Overall, the PVA film’s cell viability was improved by the addition of OsCuONPs.

### 3.10. Assay for Blood Compatibility (Haemolysis)

Blood compatibility testing was conducted on the produced plant-mediated OsCuONP and POsCuONP composite films, and the results are described in Table 4. Generally, the process of hemolysis involves the breaking down of red blood cells, which releases hemoglobin into the bloodstream and other bodily fluids. Any material or substance that comes into contact with blood must take hemolysis into account in view of biomedical applications. The results of the study demonstrated that the % of hemolysis values observed for OsCuONPs and POsCuONPs-3 were found to be 25.72% and 30.02%, respectively. This suggests that the exposure of OsCuONPs and POsCuONPs-3 films showed an acceptable lower degree of hemolysis for all three tested samples. Among the OsCuONPs, doped PVA composite films showed a lower degree of hemolysis, with a value of 25.72%, indicating that approximately 30.02% of the blood cells were lysed. The fact that no hemolytic effect was seen in any of the films under test is indicative of their biocompatibility and compatibility. A potential reason for the reduced hemolysis rate is the very hydrophilic properties of the polymer matrix that makes up the substance [65,66,67].

The hydrophilic nature of these polymers may have reduced their interactions with red blood cells (RBCs), which might explain why they were able to avoid disrupting RBCs. The material’s strong hydrophilicity, which reduces the interactions between the polymer and the red blood cells (RBCs), is the reason for the low hemolytic index of the Ch/CNP (0) film [66]. The result is a reduction in RBC disruption [66]. Consequently, these composite films are highly suitable for use in tissue engineering and wound-healing applications.

### 3.11. Wound-Healing Scratch Assay (Cell Migration)

A scratch-healing experiment allows one to monitor and quantify the process of cell migration and growth towards the center of a “wound gap” that is created when one scratches a cell monolayer [68,69]. The impact of various wound-encasing materials on the rate of closure of wounds in a cell monolayer may be assessed using the wound-healing scratch test [70]. Multiple factors can influence the pace of gap repair, contingent upon their impact on cellular motility and growth. To determine if the nanocomposite films were safe for the host tissues, we used a wound-healing scratch test to evaluate the biocompatibility of PVA and OsCuONP-doped PVA composites. Researchers utilized the scratch test to investigate in vitro wound-healing mechanisms, encompassing cell migration and proliferation, aimed at repairing skin structure [71]. Copper plays an intricate function in many different types of cells, making it one of the bioactive NPs. Furthermore, copper is an important agent throughout the whole wound-healing process, since it regulates several cytokines and the growth factor’s activity mechanisms.

**Figure 10:**
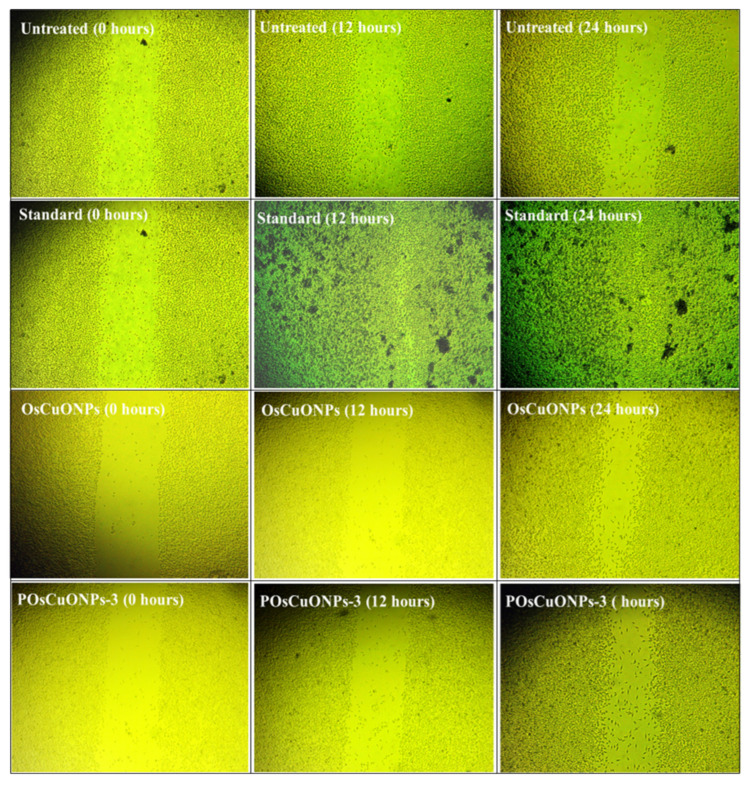
Wound-healing scratch assay images of OsCuONPs and POsCuONPs-3 composite films. **Note:** The National Centre for Cell Science (NCCS) Pune, India, provided human cervical cancer cell lines (HeLa), human breast cancer cell lines (MCF-7), human triple-negative breast cancer cell lines (MDAMB-231), and L929 fibroblast cell culture.

Figure 10 shows the images of the scratched region for the OsCuONPs and POsCuONPs-3 composite films taken at 0, 12, and 24 h. Cell migration and wound-closure studies after 24 h are shown in Table 5 and Table 6. The results of the comparison between the conventional medication and OsCuONPs are shown in Figure 9, where the latter demonstrated a cell migration activity of around 42.93%. Statistical comparisons with OsCuONPs and the gold standard medicine in Table 5 and Table 6 suggest that cell migratory activity was significantly boosted after the PVA integration of plant-mediated nanoparticles. Quantitative research verified that there was no decrease in cell migration activity when OsCuONPs were added to PVA.

The composite films composed of POsCuONPs-3 demonstrated their suitability as biomaterials after 24 h, when more than 70% of the gaps had been filled. Cell attachment and spreading were enhanced by OsCuONPs, leading to a decrease in gaps. Similar results were observed by Rezvanian et al. who prepared PVA/moringa leaf extract/graphene-oxide films for wound-healing applications and investigated cell-migration images at 0 to 6 h. They observed cells that migrated to the scratched areas with respect to time. The study showed that higher compositions (PVA/MOL-05/GO-005) exhibited higher activity, as in our study, in which a higher concentration showed a higher migration after 24 h [72]. The present study is in line with that of Asmaa et al., who investigated the lepidium sativum/PVA electrospun nanofibers prepared for wound dressing. They observed that 35% of the scratch area was covered in control and a relatively higher effect was observed in the PVA and PVA–polynanofibers. The result showed nearly 45% of the wound underwent closure. Overall, the authors confirmed that incorporating poly and TEE into the nanofibers enhanced the cell migration in the in vitro wound model [73]. The findings were highly concordant with those of Robin Augustine et al. [74]. Overall, it was found that biosynthesized silver (bAg) nanoparticles with PVA membranes helped cells move and close up a wound model in a lab, but higher concentrations slowed down wound healing. Research using silver nanoparticles (containing high-valence silver–pyridoxine complexes) by Rangasamy et al. also revealed comparable results [75].

According to their assertion, the enhanced migration and proliferation of keratinocytes was due to the mitogen-activated protein kinase (MAPK) pathway. Zangeneh has conducted effective studies on Cu-NPs using F. vulgaris leaf extracts in the bio-reduction of CuSO_4_ solutions. The authors confirmed that applying a Cu-NP ointment to a skin wound significantly improved the following wound-healing parameters: vessel density, wound contracture, hydroxyl proline, hexuronic acid, hexosamine, fibrocytes/fibroblast rate, and fibrocytes; while significantly decreasing total cells, wound area, lymphocytes, and neutrophils [76]. Another study used licorice aqueous extract to produce Cu-NPs. The efficacy of a hydroxypropylmethylcellulose gel impregnated with PHT and Cu-NPs in healing rat excisional wounds was assessed in vivo. Furthermore, a real-time polymerase chain-reaction analysis revealed that lesions treated with PHT-loaded Cu-NPs exhibited a considerable upregulation of dermal procollagen type I expression and a marked downregulation of the inflammatory JAK3 expression compared to the control group [77].

## 4. Conclusions

In the present study, OsCuONP-doped PVA (POsCuONPs) composite films were fabricated and characterized for wound-healing applications. The findings of the study showed that the addition of OsCuONPs to PVA significantly improved the physicochemical properties of the composite films, including improved mechanical and thermal properties and an evenly dispersed morphology. The results of the X-ray diffraction and WCA measurement showed that the prepared composite films were crystalline in nature, and the hydrophilicity of the composite films decreased (60.89° to 89.62°) with an increase in the concentration of OsCuONPs in the PVA matrix. The study also showed that films were nontoxic in nature. The hemolytic effect was not seen, and a wound-healing study revealed that after 24 h, 70% of the gaps had been filled in the POsCuONP-based composite films, indicating their applicability as biomaterials. The study’s overall findings validate the use of prepared composite films as biomaterials in wound-healing applications.

## Figures and Tables

**Table 1:** Composition table of PVA and OsCuONP-doped POsCuONP composite films.

Sample Code	PVA (g)	OsCuONPs (g)
PVA	2	0
POsCuONP-1	1.98	0.02
POsCuONP-2	1.96	0.04
POsCuONP-3	1.94	0.06

**Table 2:** Mechanical properties of PVA and PPCuONP composite films.

Sample Code	Thickness (mm)	Tensile Strength (*T*_s_) in MPa	Young’s Modulus (*Y*_m_) in MPa	Elongation at Break (%)
PVA	0.09	21.705	24.483	394.327
POsCuONPs-1	0.06	22.268	827.684	34.139
POsCuONPs-2	0.05	25.493	1032.642	41.714
POsCuONPs-3	0.07	32.502	1128.362	75.168

**Table 3:** The IC_50_ values of the test compounds for the L929 cell line for 24 h treatment.

Sample Name	L929 Cell Line ** IC_50_ (in µg/mL) 24 h
Untreated	100
Cisplatin	10.21
CuONPs	58.34
POsCuONPs-3	66.65

** For IC_50_ values outside of the experimental concentration range taken, the values provided above are only expected values; re-testing may be required at the higher/lower concentration range for confirmation.

**Table 4:** Percentage of hemolysis of OsCuONPs and POsCuONPs-3 composite films.

Sl. No.	Samples	% Haemolysis
1	OsCuONPs	25.72 ± 0.1078
2	POsCuONPs-3	30.02 ± 0.0904

**Table 5:** Cell-migration study of OsCuONPs and POsCuONPs composite films.

Sl. No.	Samples	Duration in h	Cell Migration in µm
1	Untreated	12	6.70
		24	5.45
2	Standard Ascorbic acid (5 µg)	12	45.40
		24	22.10
3	OsCuONPs (15 µg)	12	13.17
		24	19.26
4	POsCuONPs-3 (15 µg)	12	12.53
		24	20.60

**Table 6:** Wound-closure study after 24 h.

Sl. No.	Samples	Percentage of Wound Closure (%)
1	Untreated	22.80
2	Standard Ascorbic acid (5 µg)	93.40
3	OsCuONPs (15 µg)	42.93
4	POsCuONPs-3 (15 µg)	47.19

## Data Availability

The authors declare that the data supporting the findings of this article will be made available upon reasonable request, subject to ethical considerations.
