# Correction: Pattadakal et al. Poly(vinyl alcohol) Nanocomposites Reinforced with CuO Nanoparticles Extracted by *Ocimum sanctum*: Evaluation of Wound-Healing Applications. *Polymers* 2025, *17*, 400

**DOI:** 10.3390/polym18111385

**Published:** 2026-06-03

**Authors:** Shrishail Pattadakal, Vanita Ghatti, Sharanappa Chapi, Vidya G., Yogesh Kumar Kumarswamy, M. S. Raghu, Vidyavathi G. T., Nagaraj Nandihalli, Deepak R. Kasai

**Affiliations:** 1 Department of Chemistry, Faculty of Engineering and Technology, Jain University, Jakkasandra, Bengaluru 562112, Karnataka, India; shrish33jack@gmail.com (S.P.); vanitaghatti@gmail.com (V.G.); k.yogeshkumar@jainuniversity.ac.in (Y.K.K.); 2 Department of Physics, B.M.S. College of Engineering, Bengaluru 560019, Karnataka, India; 3 Department of Chemistry, Dayananda Sagar College of Engineering, SM Hills, Kumaraswamy Layout, Bengaluru 560011, Karnataka, India; vidyagopi5@gmail.com; 4 Department of Chemistry, New Horizon College of Engineering, Bengaluru 560103, Karnataka, India; raghuhassan2009@gmail.com; 5 Department of Chemistry, RNS Institute of Technology, Rajarajeshwari Nagar, Bengaluru 560098, Karnataka, India; vidyavathigt@gmail.com; 6 Critical Materials Innovation Hub, Ames National Laboratory, U.S. Department of Energy, Iowa State University, Ames, IA 50011, USA; nagaraj.nandi001@umb.edu

**Text Correction**

There was an error in the original publication [1]. A correction has been made to Section 2.2.5 to accurately reflect the instrumentation used. The morphology of the composite films was examined using an SEM (Carl ZEISS Sigma FE-SEM from Carl Zeiss AG, Oberkochen, Germany). The instrument was previously reported as JEOL JSM-6360. This correction does not affect the results or conclusions of the study.

In the abstract and conclusion, the water contact angle range was originally stated as 60.89° to 89.62° and has now been revised to 60.89° to 92.33°. There was a typographical inconsistency where CuONPs were mistakenly written as CuNOPs in some parts of the manuscript. This correction reflects an accurate representation of the experimental data and does not affect the overall interpretation or conclusions of the study.

**Error in Figure**

During the final review of our manuscript [1], we identified an error in Figures 1, 8 and 10, in which the x-axis of the UV–visible spectrum figure was mistakenly labelled as wavenumber instead of wavelength, and the image of POsCuONPs-3 and the Standard (0 h) panel, respectively, were inadvertently duplicated. The corrected versions of Figure 1, Figure 8 and Figure 10 are provided below.

The authors state that the scientific conclusions are unaffected. This correction was approved by the Academic Editor. The original publication has also been updated. We apologize for any confusion this may have caused.

## Figures and Tables

**Figure 1:**
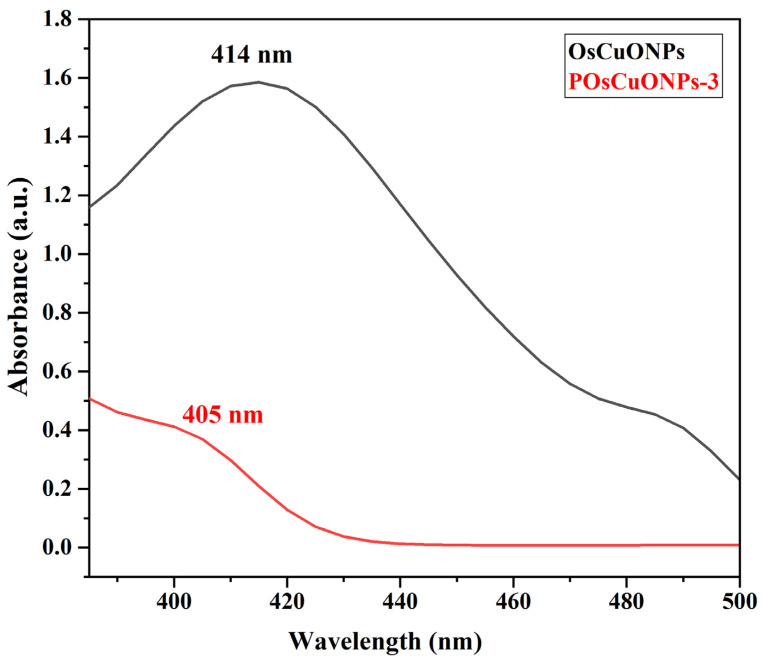
UV–visible spectra of OsCuONPs and POsCuONPs nanocomposite films.

**Figure 8:**
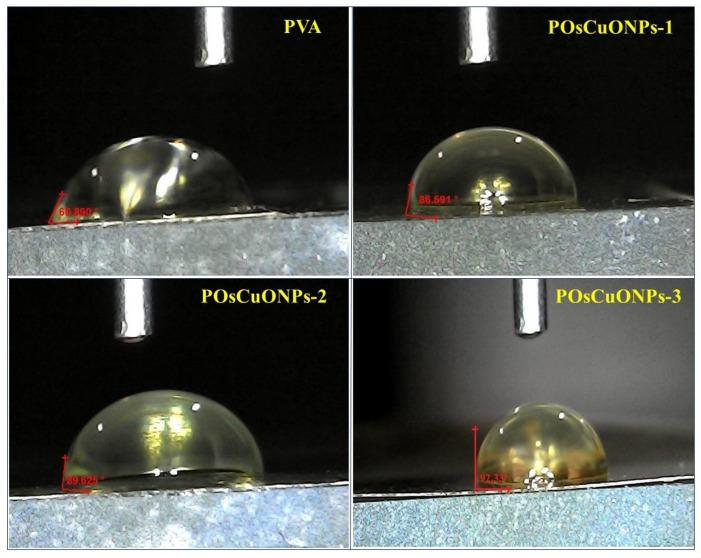
Contact-angle measurement of pure PVA and POsCuONPs composite films.

**Figure 10:**
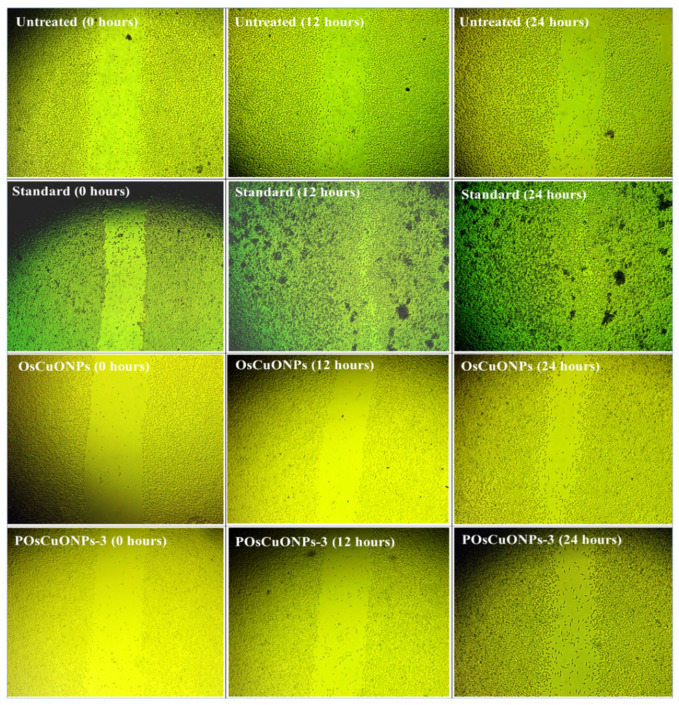
Wound-healing scratch assay images of OsCuONPs and POsCuONPs-3 composite films. **Note:** The National Centre for Cell Science (NCCS), Pune, India, provided human cervical cancer cell lines (HeLa), human breast cancer cell lines (MCF-7), human triple-negative breast cancer cell lines (MDAMB-231), and L929 fibroblast cell culture.
